# Supplementary material for: Complexome profiling of the Chlamydomonas psb28 mutant reveals TEF5 as an early PSII assembly factor
Source: Plant Cell. 2025 Mar 17;37(6):koaf055. doi: 10.1093/plcell/koaf055 (PMC12164590; doi:10.1093/plcell/koaf055)
Supplement: koaf055_Supplementary_Data [file koaf055_supplementary_data.zip › psb28_interactive.zip!/plots/intensity/SGA1_Cre01.g005150.t1.1.html]

|  |  |
| --- | --- |
| **Identifier** | Cre01.g005150 |
| **Trivial name** | SGA1 |
| **Euclidean distance** | 32796.79 |
| **Average intensity sum (WT)** | 2151.6 || **Average intensity sum (Mut)** | 3228.8 |
| **Adjusted euclidean distance** | 10.16 |
| **MapMan annotation** | amino acid metabolism.synthesis.serine-glycine-cysteine group.glycine.serine glyoxylate aminotransferase amino acid metabolism.synthesis.central amino acid metabolism.alanine.alanine-glyoxylate aminotransferase PS.photorespiration.aminotransferases peroxisomal |
| **p-value** (Welch test) of intensity sums | 0.20577 |
